# Data and models from multi-model inference of non-random mating from an information theoretic approach

**DOI:** 10.1016/j.dib.2019.104969

**Published:** 2019-12-10

**Authors:** Antonio Carvajal-Rodríguez

**Affiliations:** Departamento de Bioquímica, Genética e Inmunología, Universidad de Vigo, 36310 Vigo, Spain

**Keywords:** Mating tables, Mate choice, Mate competition, Sexual selection, Assortative mating

## Abstract

This is a co-submission with Multi-model inference of non-random mating from an information theoretic approach [1]. These data corresponds to the complete simulated data set jointly with the set of models defined for analysing the data. The simulated data set was obtained using the program MateSim [2].

The simulated cases correspond to one-sex competition and mate choice models. For each simulation run, the population frequencies (premating individuals) and the sample of 500 mating pairs were generated randomly for a hypothetical trait with two classes at each sex.

Some datasets represent larger population size species (*n* = 10 000) and the mating process was represented as a sampling with replacement, and the population frequencies were constant over the mating season. The minimum phenotype frequency (MPF) allowed was 0.1.

Five different model cases were simulated, namely random mating, female competition with mate choice (with independent or compound parameters) and male competition with mate choice (with independent or compound parameters). Each case was simulated 1000 times.

Other datasets represent monogamous species (with large or small population size) and the mating process was without replacement (from the point of view of the available phenotypes).

These data sets were used to test the performance of the multi-model inference methodology proposed in [1]. The data may be useful for testing any new/old statistics for measuring sexual selection or assortative mating patterns.

**Table:** Specifications Table

Subject	Ecology, Evolution, Behaviour and Systematics
Specific subject area	Mate competition and mate choice generate non-random mating that can be detected as sexual selection and/or assortative mating in the mating tables.
Type of data	Table Model
How data were acquired	Software MateSim Software InfoMating
Data format	Raw
Parameters for data collection	Population size, sample size, mating behaviour (monogamy vs polygamy), mate competition, mate choice.
Description of data collection	Simulation with MateSim. Model definition with InfoMating.
Data source location	Institution: Universidad de Vigo City/Town/Region: Vigo Country: Spain
Data accessibility	Repository name: Zenodo Data identification number: 10.5281/zenodo.3497325 Direct URL to data: Simulated data: http://doi.org/10.5281/zenodo.3497325 Models: http://doi.org/10.5281/zenodo.3492107
Related research article	Antonio Carvajal-Rodríguez, Multi-model inference of non-random mating from an information theoretic approach, Theoretical Population Biology, Under Revision

**Table:** 

**Value of the Data** • The data represent the effect of mate competition and choice for discrete traits under different controlled conditions. • Researchers interested in sexual selection and/or assortative mating. • The data can be used to test different estimation methods of sexual selection and assortative mating or to study how different behavioural and demography conditions affect to the pattern in the data

## 1 Data description

There are two different types of data, first type corresponds to simulated data and second type corresponds to the description of various sets of mating models.

1) Simulated data

In the Simulated_Data zip file there are 17 data folders corresponding to different mating scenarios. Each folder includes 1000 files that correspond to different Monte Carlo runs of the same mating scenario.

The name of each folder gives the full information about the mating scenario. For example the name:

i) Out_MateSim_0_a1_driftnonU_sample500_N10000_MPF0.1:
  Corresponds to mating with replacement (polygamous populations or monogamous with large population size) and a random mating model (model 0 with *a* = 1). The population frequencies are different between runs (drift) and non-uniform (nonU). The sample size is 500 matings sampled from a population of size *N* = 10 000 (sex ratio is 1:1 by default) and the minimum phenotype frequency (MPF) in the population was 0.1.

ii) Out_MateSim_6_ac2-3_driftnonU_sample500_N10000_MPF0.1:
  Stands for mating with replacement and MateSim program model 6 that implies female competition with parameter *a* = 2 and mate choice with parameter *c* = 3. The population frequencies are different between runs (drift) and non-uniform (nonU). The sample size is 500 matings from a population of size *N* = 10 000 individuals (sex ratio is 1:1 by default), and the minimum phenotype frequency (MPF) in the population was 0.1.

iii) Out_MateSim_massEM_6_ac2-3_driftnonU_sample500_N10000_MPF0.1:
  The same scenario as ii) but with mass-encounters (without replacement) instead of individual-encounters. See Refs. [2,3] for extensive explanation about individual or mass-encounters.

iv) Out_MateSim_massEM_8_ac2-3_driftnonU_sample50_N10000_MPF0.1:
  Mass-encounter model 8 with female competition (*a* = 2) plus choice compound-parameter model (*c* = 3). Sample size is 50 matings from a population of size *N* = 10 000.

The 17 data folders correspond to:

Five models (M0, M6, M7, M8 and M9) with sample 500 and N = 10 000: The item i) above corresponds to M0 while the item ii) correspond to M6; cases M7, M8 and M9 are similar to these items changing the notation for the model number, i.e. Out_MateSim_7_ instead of Out_MateSim_6_, and so on.

The model M7 is the same as M6 but for males (male competition plus mate choice). Similarly M9 is the same as M8 but for males. Three more scenarios with models M0, M6, M8 as before but with sample size 50 instead of 500.

Now the mating is changed to be mass-encounter without replacement again with models M0, M6, M8. The cases for M6 and M8 with sample size 500 were noted in iii) and iv) items. There were three more models with sample size 50. Thus, at this point we have 14 different scenarios. The last three up to 17, correspond to mass-encounter scenario for the models M0, M6 and M8 with population size *N* = 200 and sampling the whole population (sample = 100 pairs).

2) Model set

The second kind of data correspond to the set of models assayed with the program InfoMating [1]. There are two different sets of models in these data:

2-1) Simulation model set
  The file Simulations_Model_Set corresponds to the set of models assayed with the simulated data set.

2-2) Empirical model set
  The file Empirical_Model_Set corresponds to the set of models assayed with the empirical data from Ref. [4].

## 2 Experimental design, materials, and methods

1) The Simulated data were obtained with the program MateSim [2] under the specifications given in the Data Description section. Specifically, the command line arguments to obtain the 1000 files explained in i) in the Data Description section (folder Out_MateSim_0_a1_driftnonU_sample500_N10000_MPF0.1) was:

- continuous 0 -numfiles 1000 -femtypes 2 -maletypes 2 -N 10 000 -model 0 -matings 500 -uniform 0 -drift 1 -selfactor 2 -choicefactor 3
  Note that the competition factor (selfactor) and choice factor values are irrelevant since we defined the model 0 that implies random mating.
  Similarly, to obtain the 1000 files explained in ii) in the Data Description section (folder Out_MateSim_6_ac2-3_driftnonU_sample500_N10000_MPF0.1) the arguments were:

- continuous 0 -numfiles 1000 -femtypes 2 -maletypes 2 -N 10 000 -model 6 -matings 500 -uniform 0 -drift 1 -selfactor 2 -choicefactor 3
  For the files specified in item iii) i.e. mass-encounters the arguments were:

- continuous 0 -numfiles 1000 -femtypes 2 -maletypes 2 -N 10 000 -model −60 -matings 500 -uniform 0 -drift 1 -selfactor 2 -choicefactor 3
  For the item iv)

- continuous 0 -numfiles 1000 -femtypes 2 -maletypes 2 -N 10 000 -model −80 -matings 500 -uniform 0 -drift 1 -selfactor 2 -choicefactor 3
  Similarly for the remaining scenarios.

2) Model sets

The model sets were automatically provided by the program InfoMating [1]. For example, to generate all the models available in the program for the first data file in the Out_MateSim_6_ac2-3_driftnonU_sample500_N10000_MPF0.1 folder, the command line arguments were:

InfoMating -input MateSim_D_M6_ac2-3_driftnonU10000_1.txt -All 1.

Where ‘MateSim_D_M6_ac2-3_driftnonU10000_1.txt’ is the name of the first file in the folder and the tag -All 1 indicate that we desire to test all available models.

## References

[1] Carvajal-Rodríguez A. (2020). Multi-model inference of non-random mating from an information theoretic. Theor. Popul. Biol..

[2] Carvajal-Rodríguez A. (2018). MateSim: Monte Carlo simulation for the generation of mating tables. Biosystems.

[3] Carvajal-Rodríguez A. (2019). A generalization of the informational view of non-random mating: models with variable population frequencies. Theor. Popul. Biol..

[4] Cruz R., Rolán-Álvarez E., García C. (2001). Sexual selection on phenotypic traits in a hybrid zone of Littorina saxatilis (Olivi). J. Evol. Biol..

